# Habitual stone-tool-aided extractive foraging in white-faced capuchins, *Cebus capucinus*

**DOI:** 10.1098/rsos.181002

**Published:** 2018-08-22

**Authors:** Brendan J. Barrett, Claudio M. Monteza-Moreno, Tamara Dogandžić, Nicolas Zwyns, Alicia Ibáñez, Margaret C. Crofoot

**Affiliations:** 1 Cognitive and Cultural Ecology Group, Max Planck Institute for Ornithology, Radolfzell, Germany; 2 Department of Human Behavior, Ecology, and Culture, Max Planck Institute for Evolutionary Anthropology, Leipzig, Germany; 3 Department of Human Evolution, Max Planck Institute for Evolutionary Anthropology, Leipzig, Germany; 4 Animal Behavior Graduate Group, University of California, Davis, CA, USA; 5 Department of Anthropology, University of California, Davis, CA, USA; 6 Smithsonian Tropical Research Institute, Balboa, Ancón, Panamá, Republic of Panamá; 7 Department of Anthropology, University of Pennsylvania, Philadelphia, PA, USA; 8 Estación Científica COIBA-AIP, Ciudad del Saber, Clayton, Panamá, Republic of Panamá; 9 Jadwin Av. 108B, Gamboa, Republic of Panamá

**Keywords:** tool use, *Cebus capucinus*, extractive foraging, evolutionary anthropology, Coiba, primatology

## Abstract

Habitual reliance on tool use is a marked behavioural difference between wild robust (genus *Sapajus*) and gracile (genus *Cebus*) capuchin monkeys. Despite being well studied and having a rich repertoire of social and extractive foraging traditions, *Cebus* sp. rarely use tools and have never been observed using stone tools. By contrast, habitual tool use by *Sapajus* is widespread. We review theory and discuss factors which might explain these differences in patterns of tool use between *Cebus* and *Sapajus*. We then report the first case of habitual stone tool use in a gracile capuchin: a population of white-faced capuchins (*Cebus capucinus imitator*) in Coiba National Park, Panama who habitually rely on hammerstone and anvil tool use to access structurally protected food items in coastal areas including *Terminalia catappa* seeds, hermit crabs, marine snails, terrestrial crabs and other items. This behaviour has persisted on one island in Coiba National Park since at least 2004. From 1 year of camera trapping, we found that stone tool use is strongly male-biased. Of the 205 camera trap days where tool use was recorded, adult females were never observed to use stone tools, although they were frequently recorded at the sites and engaged in scrounging behaviour. Stone tool use occurs year-round in this population; over half of all identifiable individuals were observed participating. At the most active tool use site, 83.2% of days where capuchins were sighted corresponded with tool use. Capuchins inhabiting the Coiba archipelago are highly terrestrial, under decreased predation pressure and potentially experience resource limitation compared to mainland populations—three conditions considered important for the evolution of stone tool use. White-faced capuchin tool use in Coiba National Park thus offers unique opportunities to explore the ecological drivers and evolutionary underpinnings of stone tool use in a comparative within- and between-species context.

## 1. Introduction

Extractive foraging permits many generalist species to access structurally protected resources. It requires both physiological specializations and/or cognitive traits that aid in resource manipulation and extraction. Extractive foraging may permit organisms to outcompete species which lack such adaptations and may be of great importance in ecologically stressful periods as a fallback foraging strategy [1]. It is also important to the ecological success and evolutionary history of many primates.

Tool use is a taxonomically widespread [2,3] form of extractive foraging which may permit access to novel resources, expand diet breadth and may be useful to more efficiently or safely access existing resources. Comparative studies of tool use are key to understanding the ecological and social factors that drive its evolution within the primate lineage. In primates, the cognitive skills required for tool use was probably selected for alongside social and ecological intelligence [4] and generalized problem-solving [5] (but see [6] for exceptions to this in other taxa). Once tool use evolves it may create interesting eco-evolutionary feedbacks via gene-culture coevolution and/or cultural niche construction that are probably important in hominin evolution [7–9]. The cultural transmission of toolkits and associated behaviours is of central importance to human evolution; it is key to our success as the most widely dispersed vertebrate on Earth.

Comparative studies of stone tool use in non-human primates are of particular interest to palaeoanthropologists as it provides a model of the possible tool use behaviours that led to the emergence of earliest known stone tool production in hominins. Percussive techniques like those observed in non-human primates may have been the precursor to the earliest hominin stone tool making around 3 Ma [10–14]. Thus, a better understanding of tool use in non-human primates helps us to validate and critically evaluate our interpretations of the fossil and archaeological records [15]. Further, comparative studies of tool use in extant primates allow us to better interpret and understand what early lithic technologies looked like, how they may have been used by ancient hominins, as well as how site preservation and visibility (e.g. [10]) affect our ability to interpret the fossil record.

### 1.1. Differences in tool use between robust and gracile capuchins

Among capuchin monkeys, habitual reliance on tools, and stone tools in particular, has been considered a distinguishing feature of the larger-bodied robust capuchins (*Sapajus*) from the smaller gracile capuchins (*Cebus*) since their divergence 6.5 Ma [16]. Stone tool use has been observed in the wild in all well-studied robust capuchin species including black-striped capuchins, *Sapajus libidinosus* [17–19]; yellow-breasted capuchins, *S. xanthosternos* [20]; blonde capuchins, *S. flavius* [21]; black-horned capuchins, *S. nigritus* [22]; and black-capped capuchins, *S. apella* [23] (based off taxonomic reclassifications [16,24] and reviews by Ottoni & Izar [25] and Garber *et al*. [26]). Other forms of tool use, especially the use of sticks to dig and probe, have also been reported in a population of *S. libidinosus* [27,28] and more anecdotally or with single observations in *S. apella* [29], *S. flavius* [30] and *S. macrocephalus* [31].

Using one liberal definition of tool use adapted from Chevalier-Skolnikof [32] (‘the use of one unattached object to effect a change in another’), many of the combinatorial actions that are habitual components of the behavioural repertoire of *Cebus* would qualify as tool use ([33] ch. 7 for a review). This includes common foraging behaviours like pounding and scrubbing fruits and animal prey on rocks or branches [34–37], as well as their use of branches as fulcrums, and breaking sticks in aggressive displays [38]. Although there are several reports of *Cebus* using sticks as clubs, most are one-time observations [39]; whether this behaviour is intentional or exploratory is debated [33]. This contrasts with Beck’s [40] more commonly accepted definition where three criteria must be satisfied to qualify as tool use: the potential tool cannot be part of the animal, must not be attached to the surrounding environment and must be manipulated to achieve a useful outcome (but see [41] for inconsistencies regarding use of this definition).

Using this more stringent and widely used definition, tool use is rarely observed in gracile capuchins. Some social groups of white-faced capuchins, *Cebus capucinus imitator*, use leaves as sheathes to process stinging *Automeris* caterpillars and *Sloanea* fruits [35,42]. White-fronted capuchins, *C. albifrons* have been observed to use leaves as sponges for drinking [43] while white-faced capuchins do the same with leaves, dried wasp nests and fruits [42]. However, while tools may be helpful for acquiring these resources, they are not required. No examples of tool use have been reported in wedge-capped capuchins *C. olivaceous* or many of the recently reclassified [24,44] and less well-studied species (i.e. *C. yuracus, C. unicolor, C. kaapori, C. versicolor, C. cesarae, C. leucocephalus and C. brunneus*).

Habitual use of tools, and stone tools in particular, is considered one of the defining behavioural differences between *Cebus* and *Sapajus* [45]. The near absence of tool use in gracile capuchins is puzzling as they are otherwise highly innovative [42] and have a wide array of social [46] and foraging traditions [35,47,48] which are socially learned [36,49]. In addition, many physiological and social traits which are associated with tool use, including tolerance of close observation by conspecifics [34,50], exploratory behaviour [42,51] and dextrous hands [52–54] are present in both gracile and robust capuchins.

### 1.2. Hypotheses explaining the origins and/or maintenance of tool use

Stone tool use has been observed in only three genera of wild primates: chimpanzees (*Pan troglodytes*) [55,56], robust capuchins (*Sapajus* spp.) [25] and macaques (*Macaca fascicularis*) [57,58]. Within each of these groups, however, significant variation in tool use behaviour exists. This variation warrants further explanation. There are four commonly evaluated hypotheses to explain the presence and absence of tool use in animal populations. Fox *et al*. [59] synthesized three hypotheses for why primates use tools while another was articulated by Rutz *et al*. [60].

The *necessity hypothesis* suggests that ecological need drives stone tool use. The original formulation of this hypothesis presupposes that resources are distributed in a manner that favours scramble competition (which may be driven by high population densities) and predicts that tool use will be common in populations with relatively less favourable energy budgets [59]. The necessity hypothesis primarily pertains to the evolutionary origins of tool use, but could also account for its maintenance. Subsequent formulations of this hypothesis have focused on an overall reduction in resource availability and/or seasonally limited access to food as the causal factor driving tool use innovations [61,62]. However, current data on environmental seasonality and resource fluctuations may not reflect the historic environmental conditions experienced by species (or populations) when tool use traditions emerged. These results must therefore be interpreted with caution.

The *opportunity hypothesis* posits that increased availability of, and encounter rates with, tool material and appropriate food resources drives tool use [59]. This hypothesis suggests that tool-using populations simply live in conditions where they are more likely to encounter materials for tool use and resources which require tools for extraction. It does not make any assumptions or predictions about differences in resource distributions or foraging efficiency between tool-using and non-tool-using populations.

The *relative profitability hypothesis* was originally articulated in studies of tool use in New Caledonian crows [60,63]. It suggests that tool-aided foraging behaviours are used when they have a better energetic return rate for accessing structurally protected or embedded foods than non-tool-assisted foraging techniques. This differs from the necessity hypothesis in that it does not necessarily presuppose any differences in resource abundances between tool-using and non-tool-using populations or species [64]. Further, in contrast with the necessity hypothesis, the relative profitability hypothesis is primarily concerned with the maintenance of tool-using behaviours as a function of their increased economic utility, rather than their evolutionary origins.

The *limited invention hypothesis* [59] is less commonly discussed, as it is difficult (or impossible) to empirically test (but see [64]). It states that tool use may be invented rarely, or might be difficult to spread and/or maintain, and therefore the patterns of tool use we observe across species and populations are driven by neutral evolutionary processes rather than natural selection. This hypothesis predicts that instances of tool use might occur in disparate geographical areas where barriers to dispersal and cultural transmission exist and that more frequent or complicated tool use will occur under conditions where constraints on transmission are relaxed. Barriers to dispersal and cultural transmission may be social, cognitive or geographical. Fox *et al*. [59] argued that the limited invention hypothesis is supported when no evidence for the first two can be found. However, it is possible that the origins of a behaviour might be favoured by selective pressures consistent with the necessity hypothesis or historical factors (the environment an animal evolved in) consistent with the opportunity hypotheses. Maintenance may be affected by factors consistent with the limited invention hypothesis.

Addressing these four hypotheses is challenging as they are not mutually exclusive. Within-species comparisons provide support for all of them ([61,64] for reviews). Within-population support in Brazilian populations of *S. libidinosus* for the opportunity hypothesis has been found for nut cracking with stone tools at Fazenda Boa Vista [65] and using stones to dig for invertebrates and tubers at Serra da Capivara National Park [66]. Between-species comparisons of chimpanzees and bonobos did not find support for the necessity, opportunity or limited invention hypotheses and instead put forth that the ancestral ecological conditions in the Pleistocene might explain current behavioural variation [64]. Current environmental conditions may not reflect the historic ecological or social conditions that favoured the origins of tool use. The spatial scales of resource availability and territory size may differentially favour tool use in closely neighbouring populations of the same species, so habitat-wide information may not be of use to particular social groups. The temporal scales in which resource bottlenecks occur that make tool-aided extractive foraging necessary for population persistence may also not be captured on the timescale of a typical research project. For these reasons, it is important to be precise regarding if we are addressing the origins/and or maintenance of tool use and endeavour to collect data on temporal or spatial scales that are most applicable to our hypotheses.

Another challenge about addressing these hypotheses is that the spatial and temporal scales at which we explore them may exist on different levels of biological organization [67]. Also, these hypotheses probably blur the lines between the proximate–ultimate distinction [68]. The opportunity hypothesis, when viewed over evolutionary time, is a question about the historic environmental conditions in which ancestral populations evolved and whether they were conducive towards tool use. However, it is often treated as a hypothesis about proximate mechanisms: is stone tool use more likely to develop in social groups or species that are more likely to encounter stone tools in their environment? This contrasts with the necessity hypothesis and relative profitability hypothesis which focus on ultimate causation, ecological function and fitness outcomes. The limited invention hypothesis, by contrast, focuses on the proximate factors which affect the spread of an adaptive tradition which may subsequently affect fitness outcomes. This interaction between both levels of biological organization is probably important for understanding the distribution of tool use behaviour among primates.

### 1.3. Important factors in explaining variation in tool use in capuchins

Prior work has identified several factors which may explain variation in tool use among extant primates and what affects the origins and maintenance of this behaviour [18,59,61,69,70]. These factors include resource limitation due to seasonal reductions in food abundance (i.e. tool use as a fallback strategy), high abundance of nutritious, embedded foods which require tools to access, low dietary richness, availability of stones and anvil sites, increased terrestriality and low predation risk.

Sufficient abundance of embedded foods and availability of tool-making materials are necessary preconditions for stone tool use. They provide a proximate explanation for why stone tool use is present (or absent) in a particular group or species. The abundance and nutritional quality of resources are also important in addressing the relative profitability hypotheses. Resource limitation, low dietary richness, increased terrestriality and low predation risk impact the relative benefits and costs of tool use, providing an evolutionary explanation for why tool use arises and persists in some cases but not others.

The distribution of stone tool use among *Sapajus* populations supports the hypothesis that resource limitation and low dietary richness may favour the evolution of this behavioural strategy; tool-using *Sapajus* populations tend to live in drier, more seasonal areas such as the Cerrado and Caatinga of Brazil which have less primary productivity, plant richness and fruit abundance compared to populations living in the rainier, more productive, species-rich Amazon forest [69]. It has also been proposed to be a difference between gracile capuchins in central America and robust capuchins in drier regions of Brazil. However, populations of *Cebus* live in diverse tropical ecosystems including seasonal dry forests (where the majority of well-studied populations live), primary and secondary rain forests and montane cloud forests. To date, no studies have compared extractive foraging or tool use across these ecosystems within *Cebus*. The hypothesis that organisms will rely on tool use as a fallback strategy when easy to process fruits are seasonally limited [1] receives support in robust capuchins and chimpanzees [61,71]. However, populations of *C. capucinus* rely more on extractive foraging of embedded insects and become more terrestrial when resources are limited at the end of the dry season [50,72].

The relative profitability hypothesis has not been directly compared between populations of tool-using and non-tool-using capuchins. If a resource is abundant, easily encountered and of particularly high quality, perhaps the costs of learning and using tool use will be compensated for by increased nutritional returns. Structurally protected foods which require tool use might be included in the diet if other resources in the environment are sufficiently rare or are of low nutritional quality in accordance with optimal foraging models [73,74]. Additionally, resource abundance and quality is likely to interact with dietary richness, and an increased reliance on tool use might be favoured when there are a limited set of food options available. However, any hypothesis about foraging optimality in primates might be better informed by social foraging models [75]. Tool use often generates producer–scrounger dynamics, which also affects the probability that a foraging behaviour will be culturally transmitted [76]. If age or sex differences in the efficiency or proclivity to use stone tools exist, we might predict different reliance on tool use across age and sex classes and potential indirect impacts of social behaviour.

Reduced dietary richness might also explain the difference between tool-using and non-tool-using populations of *Sapajus* and *Cebus*. Groups which live in areas with fewer potential diet items, such as islands or low-quality habitats, may be forced to innovate and invest in potentially costly behavioural experimentation to make the best of a bad situation. It is also possible that differences in tool use rates might be influenced by ranging patterns. Gracile capuchins have larger home ranges than robust capuchins, on average (reviewed in [45,77]), and thus might be able to access a more diverse set of food items that do not require tool use compared to robust capuchins. However, it is also possible that groups of robust capuchins are just more capable of extracting resources out of a smaller area due to morphological specializations such as thicker enamel and larger molars [16].

Stones are needed for stone tools to be used. High availability of the necessary raw materials in the environment increases the probability that an individual will interact with them and innovate stone tool use. The importance of such opportunities for innovation is supported by within-*Sapajus* variation in stone tool use. Populations of *Sapajus* (and *Cebus*) living in the alluvial floodplains of the Amazon, for example, have less access to stones than populations living in rockier, drier habitats, and do not use stone tools [69]. However, while the availability of raw materials may drive some within-genus or within-species variation, it is unlikely to explain broader taxonomic differences in stone tool use; within any *Cebus* or *Sapajus* species’ range there exists considerable geological variation.

Increased terrestriality might explain the presence of stone tool use [70,78] consistent with the opportunity hypothesis. Individuals are more likely to encounter and wield stone tools on the ground than in the canopy. However, both *C. capucinus* and *C. albiforns* [79] can be exceptionally terrestrial, sometimes in response to seasonal fluctuations in fruit availability [72], yet, no previously studied populations of *Cebus* appear to use tools of any kind with any great frequency.

Decreased predation risk may lead arboreal species to become more terrestrial (Monteza-Moreno *et al*. [80]), thus, making them more likely to invent or use stone tools on the forest floor. However, the absence of predators might also affect ecological competition between conspecifics and heterospecifics with niche overlap. Increased within- and between-species competition may affect both population density and ranging behaviours of a candidate tool-using species and the abundance of heterospecifics with niche overlap, creating feedback loops which negatively affect species richness and abundance which may further favour the evolution of tool use. Reduced predation risk may also directly affect the probability of stone tool use by creating safe conditions where animals can afford exploratory behaviour, potentially leading to the innovation of tool use [60,81]. Stone tool use, in particular, may also directly attract predators; it is a loud, conspicuous activity that often occurs in predictable areas where resources are clustered, making tool-using individuals easy targets for predators. Stone tool use also requires concentration, thus decreasing an organism’s ability to be vigilant. Differences in predation risk may explain both within- and between-species variability in tool use.

## 2. Study site

Coiba National Park in the Gulf of Chiriquí of Panama is an archipelago encompassing nine larger islands and more than 100 islets. The main island, Coiba, lies 23 km off Panama’s Pacific coast and has been geographically isolated from the mainland for 12 000–18 000 years [82]. Coiba National Park has significant ecological variation: moist and wet forest account for 60% of its habitat types [83], and the park also includes mangroves and swamp forests. Coiba National Park receives 3500 mm of rain per year which primarily falls during a marked wet season (May–December). Coiba National Park (CNP) contains one of the largest remaining parcels of Pacific Central American forest; 75% of the park consists of mature forest, due to the island of Coiba’s historic (until 2004) role as a penal colony [84].

Prior to the penal era which began in 1919, the archipelago (including the islands which contain capuchin monkeys) was utilized and inhabited by indigenous people who probably arrived from the Azuero Peninsula as early as 250 CE and by subsequent groups from modern-day Chiriquí and Coclé [85]. Europeans first arrived in Coiba in 1551; subsequent genocide, disease and enslavement by Spanish colonists eliminated indigenous populations on the island by the end of the sixteenth century [82]. During penal colony times, up to 1000 prisoners and their guards lived in 20 prison camps, mostly along the east coast of Coiba, and including one in the northern coast of Jicarón. Currently, only two islands in CNP have any constant human occupation including a research station on Ranchería and a police station and national park visitors centre on the northeast portion of Coiba. The other islands, including Jicarón, have no recent long-term human settlement; human–wildlife interactions are rare and mostly restricted to occasional interactions with local fishermen and tourists along the coast.

Coiba National Park is a UNESCO world heritage site and is a hotspot for marine and terrestrial plant and animal endemism [83,86–90], much of which is understudied. This includes five endemic species and subspecies of terrestrial mammals: the Coiban agouti *Dasyprocta coibae*, Coiba Island or Rothschilds’ white-tail deer *Odocoileus virginianus rothschildi*, Coiba Island howler monkey *Alouatta palliata coibensis* or *Alouatta coibensis*, black-eared opossum *Didelphis marsupialis battyi* and potentially a rodent of the genus *Zygodontomys* [91].

Three of the largest islands in Coiba National Park, Coiba (50 314 ha), Jicarón (2002 ha) and Ranchería (222 ha) support populations of capuchin monkeys (*C. capucinus imitator*) (electronic supplementary material, figure S1). Observations from a 12-day survey in 1974 estimated capuchin group sizes on Coiba to be similar to mainland populations at Barro Colorado Island [84]. Later estimates [92] show similar group sizes with a mean of 10.75 individuals (range 5–16) and suggest that capuchins on Coiba Island occur at low densities. However, an increased sampling effort, different surveying methodologies (such as randomization, camera trapping and mark-recapture analysis) and recent advances in analytical approaches might yield a different estimate. Longitudinal studies in Costa Rica show larger maximum annual group size with much variance (mean = 23.20, s.d. = 9.26, range = 5–40, estimated from 20 years of data in fig. 7.1 in [93]). Our qualitative impression on Jicarón shows group sizes up to an estimated 20 individuals and at potentially high population densities. However, more systematic data collection, increased sampling effort and nuanced analysis is needed to make reliable comparisons of group size and population density, so current estimates should be considered tentative.

Capuchins living on the islands within the Coiban archipelago provide a natural laboratory for examining hypotheses about the evolutionary drivers of tool use. Populations are geographically isolated by the ocean, strongly limiting genetic flow and cultural transmission. Additionally, the three islands differ in the number of potential food items available to capuchins, following well-established patterns of species richness varying as a function of distance from the mainland and island area [94]. Plant richness on Coiba is less than what is observed in similar forests on the Panamanian mainland [95]. Transect data (Ibáñez *et al.* [96]) show that Coiba, the largest island, located in the middle of the archipelago, is home to 1001 plant species. Jicarón (the most distant island) and Ranchería, by contrast, host similarly depauperate plant communities (261 and 262 species, respectively), despite a nearly 10-fold difference in size [97]. Smaller islands are also more likely to suffer from ecological perturbations due to edge effects. These two factors combined with the highly terrestrial behaviour of capuchins in Coiba National Park (which may increase encounter rates with material for stone tool use) due to an absence of terrestrial predators on the islands [84] provides ample opportunity (and decreases potential costs) for capuchins to use stone tools.

## 3. Methods

### 3.1. Coastal surveys

In 2004, A. Ibáñez first observed capuchins using stone tools to crack open the endocarps of *Terminalia catappa*—a coastal tree from Asia which has been nativized in the Neotropics and is known commonly in Spanish as *almendro de playa* and in English as *sea almonds*. Following up on this initial report, a documentary field crew captured this nut-cracking behaviour on film in 2013 (Paul Stewart and Huw Cordey 2017, personal communication). To further document and determine the extent of this unique behavioural variant, we conducted field surveys from March 2017 to March 2018. B. Barrett and C. Monteza-Moreno surveyed the coasts of Jicarón, Coiba and Ranchería by boat to identify *T. catappa* groves. We then surveyed these islands, including the entirety of the coasts of Jicarón and Ranchería at least twice, on foot to look for evidence of tool use, including broken endocarps, hammerstones and anvils, cracked crab and bivalve shells and coconuts. We also surveyed approximately 8 km of the coast of Coiba with rocky intertidal zones and/or *T. catappa* groves.

### 3.2. Camera trapping effort

**Figure 1.**
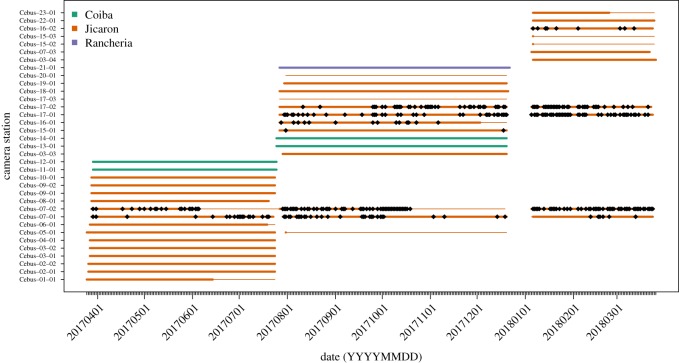
Visualization of camera trap sampling effort. Rows are camera trap locations. Smaller lines indicate length of deployment, thick lines indicate period of deployment when camera was filming and focused on tool use site (i.e. was not moved by animals, had working batteries, was not stolen or vandalized). Diamond points occur at each day where stone tool use was recorded. Colours indicate island in Coiba National Park.

To assess potential tool use sites, we deployed unbaited photo (Hyperfire HC600) and video (Ultrafire XR6) camera traps (Reconyx, Inc, WI, USA) between 25 March 2017 and 26 March 2018. Photo-trapping is advantageous for recording localized tool use for unhabituated primates in remote areas [98]. Photo camera traps were set to take a series of 10 images per trigger event with no delay between triggers, allowing us to document behavioural sequences. Video camera traps captured sequences consisting of one photo followed by 30 s of film per trigger. We deployed cameras at 35 sites on the islands of Coiba (four sites, 532 trap-nights), Jicarón (30 sites, 3466 trap-nights) and Ranchería (one site, 148 trap-nights) (figure 1). At four sites (two Coiba, one Ranchería, one Jicarón) where we found almendros and capuchins but few rocks, we set up experimental anvils and hammerstones to see if capuchins would use them.

### 3.3. Weighing and scanning tools

Graphic documentation in the field consisted of geo-located digital pictures of sites and tools, three-dimensional models of sites using geo-referenced images (Agisoft Photoscan), hand-made and digital mapping and situational sketches. The use of this set of standardized archaeological methods facilitates comparison with archaeological sites (and any others when the same protocols are used). Using a digital scale, we weighed a subset of the hammerstones (*N* = 60) used in the behavioural sequences captured by our camera traps or observed during coastal surveys (i.e. stones which were piled on broken *Terminalia* endocarps, resting on coastal driftwood). Broken hammers were refitted on site before being weighted. A set of photographs of several of these tools were taken with a goal to create three-dimensional models according to protocols established by Porter *et al*. [99].

### 3.4. Statistics

To estimate the size of stone tools and monthly rates of tool use across camera stations, we used generalized linear models (GLM) and generalized linear mixed effect models (GLMM) fit using the map2stan function in the rethinking package (v. 1.59) [100] in R (v. 3.43) [101]. map2stan is a front-end which utilizes rSTAN (v. 2.17.2), a Hamiltonian MCMC sampling engine [102]. To estimate mean stone tool size, we fit a Gamma GLM using a log link as stone tool weights are positive, non-normal and lower-bound by zero. To estimate monthly tool use rates, we used an aggregated binomial GLMM with a logit link. To analyse stone tool use rates, we analysed a subset of camera trap data where stone tool use was observed at least once. Our outcome was the number of days per month where tool use was observed at each camera station and the number of trials was the number of days per month where a camera recorded a capuchin at least once. We estimated varying intercepts for each camera station (*N* = 7), camera station deployment (*N* = 13) and unique month (*N* = 12). All data, model and graphing code for this paper can be found at this manuscript’s corresponding GitHub repository. These annual rates are a coarse, preliminary measure of stone tool use in this population and future analyses will account for inter-individual and monthly variation in tool use rate at more consistently sampled tool use sites.

## 4. Findings

### 4.1. Habitual reliance on stone-tool-aided extractive foraging in white-faced capuchins

**Figure 2.**
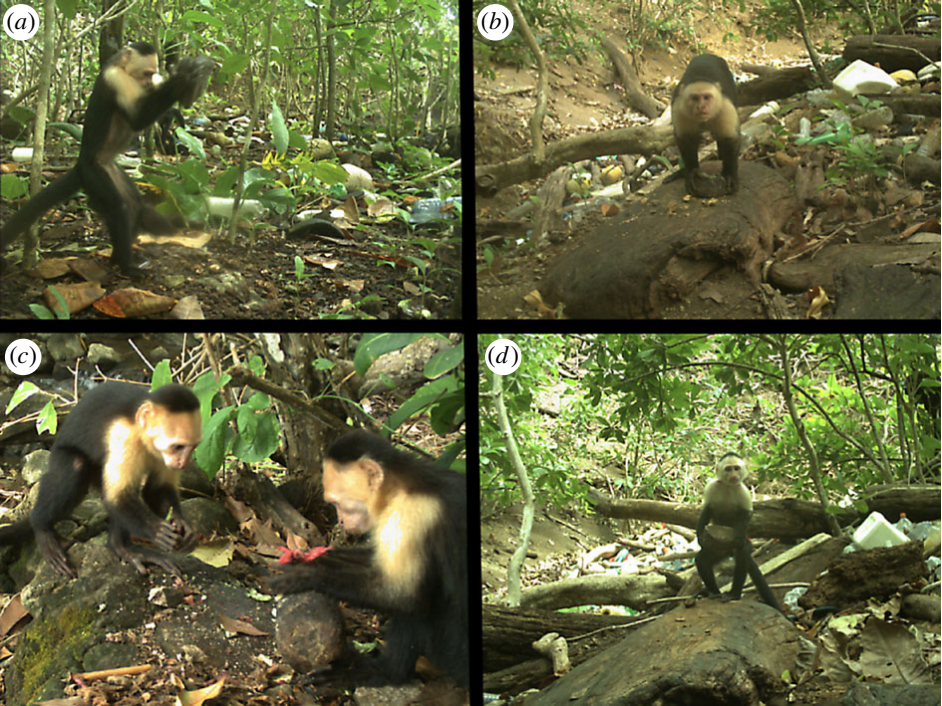
(*a*) A juvenile male capuchin uses a hammerstone to crack open a *T. catappa* endocarp on a stone anvil. (*b*) An adult male after cracking open a *T. catappa* endocarp on a wooden anvil. (*c*) Juvenile male capuchin observing an older juvenile processing *T. catappa* endocarps with a hammerstone. (*d*) Juvenile male about to process *T. catappa*—note prehensile tail used for support.

**Figure 3.**
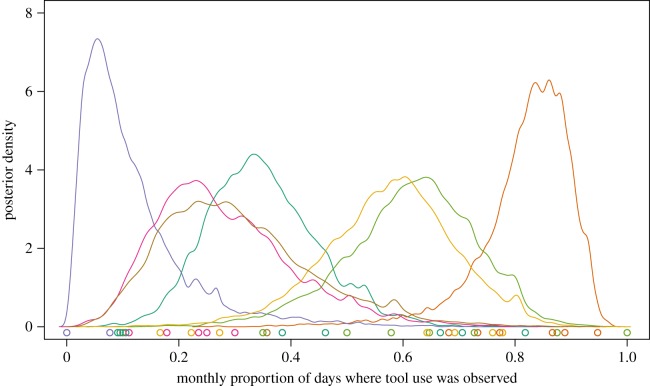
Tool use sites differed greatly in their observed usage. On average, tool use was observed at each camera site on 45.3% of days where capuchins were observed. Cameras at the most active tool use sites (orange curve on far right) recorded tool use on 83.2% of the days where capuchins were observed. Curves show posterior predictions of probability of observing tool use on each day conditional on observing a capuchin across all months. Colours correspond to camera stations. Points on *X*-axis are raw proportions.

**Figure 4.**
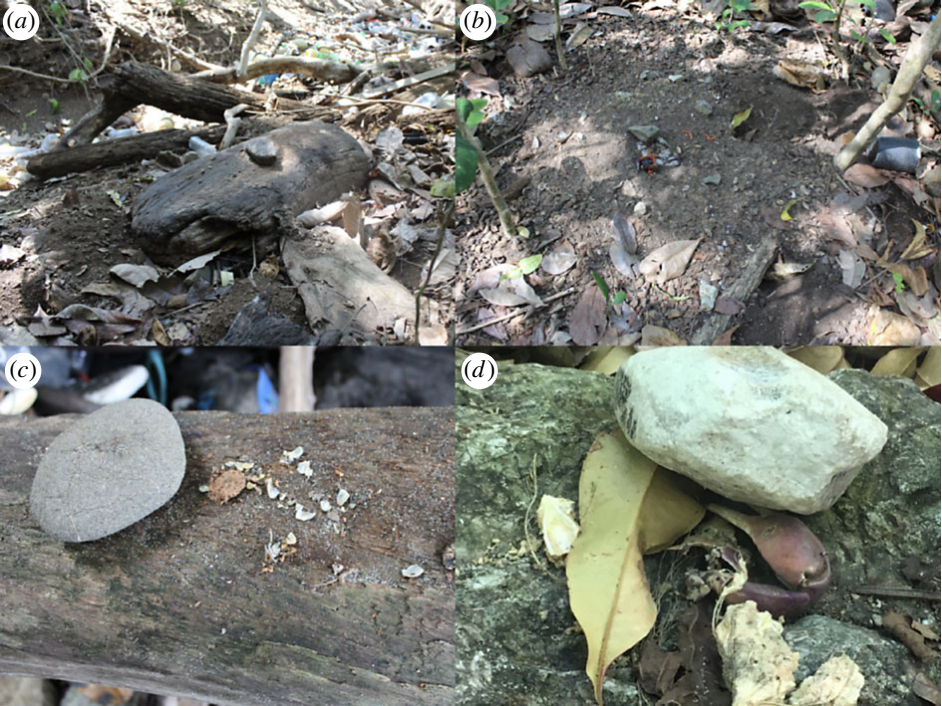
Wooden (*a*,*c*) and stone anvils (*b*,*d*) used by capuchins to process structurally protected foods. Note accumulation of processed materials including old endocarps, coconut husks and shells in (*a*,*b*). (*c*) Hermit crab exoskeleton and shell remains processed with a stone tool on dead tree branch. (*b*,*d*) Stone hammer on stone anvil with *T. catappa* endocarps and halloween crab remains. The leaf stuck under the hammer in (*d*) indicates that the food was processed shortly before discovery. Percussion impacts can be observed on the crab limb.

**Figure 5.**
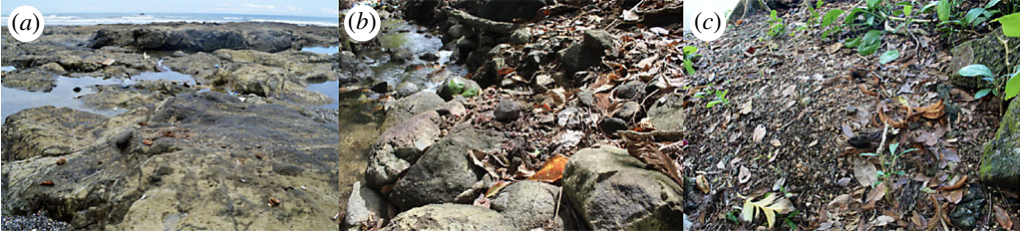
(*a*) Elusive tool use occupation in the intertidal zone that is regularly destroyed by daily tidal changes. (*b*) Medium-sized tool use occupation along stream bank that is destroyed less regularly by seasonal floods. (*c*) Large tool use occupation lying on higher ground away from streams and shore. Accumulation of tools and processed food shows potential for archaeological excavation.

We found that capuchins on Jicarón in Coiba National Park habitually use stone tools to open a variety of food resources including *T. catappa* endocarps (figure 2; electronic supplementary material, video S1), hermit crabs (figure 4*c*; electronic supplementary material, video S1) and halloween crabs (*Gecarcinus quadratus*) (figure 4*b*,*d*). Coconuts (*Cocos nucifera*) (electronic supplementary material, figure S3) and marine snails are also processed with a hammerstone and anvil, although we observe it much less frequently. At the most active tool use site, 83.2% of days where capuchins were observed corresponded with tool use (figure 3). Middens of *T. catappa* shells, coconut husks and tool shards are readily apparent at tool use sites (figures 4*b*,5*c*). Coconuts are commonly eaten by capuchins but they are almost always opened using only a stone or wooden anvil, as has been described on Coiba Island [37] and is widespread across Jicarón. At the tool use site, juveniles primarily use stones to pound coconut flesh from husk shards after they have been opened—using a hammerstone, so far, appears uncommon.

Temporary tool use occupations are also commonly observed in the intertidal and are regularly wiped out by daily changes in the tide (figure 5*a*). Small- and medium-sized occupations are located within dry stream beds (figure 5*b*) and larger accumulations of processed food lie on higher ground (figure 5*c*). This situation suggests that the joint effect of the tide and seasonal activity of the stream impact site formation processes and thereby, may affect site visibility. Further, during coastal surveys, we observed stones piled on broken clam shells at the edge of the intertidal zone, consistent with capuchins using stone tools to exploit this rich marine resource (electronic supplementary material, figure S2). In some instances, the stones were spaced in a regular pattern, at distances similar to the inter-individual distances observed in foraging capuchins. Destruction of cameras by vandals and harsh marine conditions prevented us from confirming this behaviour.

In contrast with Jicarón, our initial coastal surveys and camera trapping failed to return evidence of stone tool use by capuchins on the islands of Coiba or Ranchería (figure 1). This occurs despite the presence of the necessary lithic materials and structurally embedded foods on all three islands. Halloween crabs, hermit crabs, marine snails, coconuts and *T. catappa* occur on all islands. Abundances of *T. catappa* trees are currently being estimated, but preliminary observation suggests that they are relatively rarer on Ranchería. Groves of similar (or higher) densities occur on sections of Coiba and Ranchería where we have not observed stone tool use. Future studies will address the relative abundances within and between islands of the other two primary processed food items—halloween crabs and hermit crabs. We did not observe tool use at any of the artificial anvil sites we set up on the islands (figure 1).

### 4.2. Description of stone tools

Capuchin tool use primarily consisted of pounding food items using a hammerstone. Preliminary observations indicate that the pounding technique is direct percussion with a hard hammer (*sensu* [103], figure 2; electronic supplementary material, video S1). Food items were placed on stationary anvils of mineral or organic material (figures 3 and 4). Mineral anvils included rocky outcroppings and bedrock along stream banks, in the forest interior and in the intertidal zone (figure 5). In cases where stones were used as anvils, this technique could have produced a rebound force. Large fallen trees and logs in the forest and washed up in the intertidal zone were also used for pounding, as were the bases of trees and tree branches. In some cases, hammerstones were transported 2–3 m up into the canopy. When used as a support for pounding, wood is less likely to produce a rebound force, suggesting a use of wood as a stand-on surface to increase the accuracy of the strike or easy collection of the food item.

**Figure 6.**
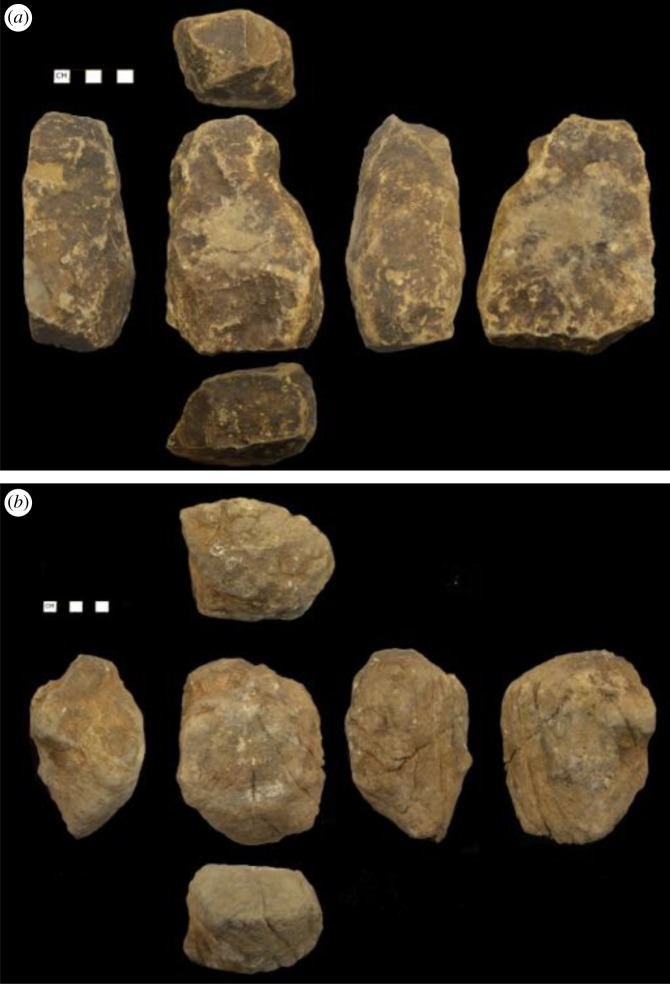
Examples of hammerstones from Jicarón sites used for nut-cracking weighing (*a*) 989 g and (*b*) 1202 g.

**Figure 7.**
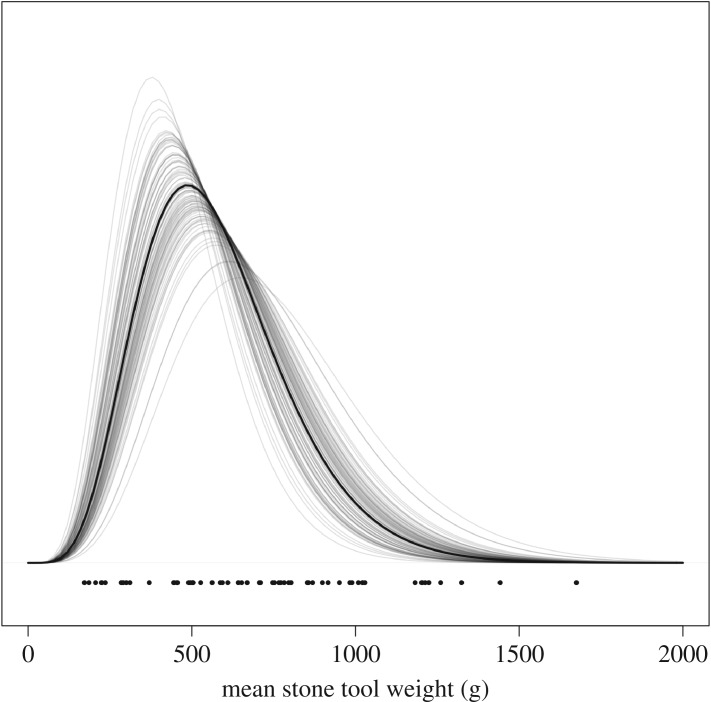
Model predictions from a gamma GLM of mean stone tool weight. Dark line is posterior mean estimate, while lighter lines are 100 randomly sampled posterior predictions to visualize uncertainty.

Capuchins primarily used cobbles collected from streams as hammerstones (figure 6). In some cases, stones from the marine intertidal and eroded hillsides were also used to process food items. A sample of the tools randomly collected on trips in July 2017, January 2018 and March 2018 from 60 tool use sites used by capuchins on Jicarón varied greatly in mass (mean = 740.7 g, s.d. 402.7, range 172–2256 g, *n* 60; figure 7). Capuchins used large stones—one weighing more than 2 kg—to process *T. catappa*, adopting a two-handed grip with a bipedal stance, and often using their semi-prehensile tails for support and leverage. By contrast, smaller items such as hermit crabs and snails were cracked with a two- or one-handed grip in a crouching position.

Based on our photographic evidence and surveys, both hammerstones (electronic supplementary material, figure S4) and food items were sometimes transported to the tool use sites (electronic supplementary material, video S1). *Terminalia catappa* endocarps, for example, were often collected multiply underneath *T. catappa* trees at the shoreline and transported to anvil sites deeper in the forest. In this population, the use of stone tools is heavily male-biased. Over the course of 1 year, we did not observe a single adult female use stone tools despite them commonly engaging in scrounging behaviour in the vicinity. Tool use in this population also generates interest and close-range observation from conspecifics, providing opportunities for social learning (figure 2*c*; electronic supplementary material, video S1).

## 5. Discussion

This is the first description of habitual stone tool use in gracile capuchin monkeys, adding *Cebus* to the list of primate genera with populations who habitually use stone tools in the wild (*Sapajus, Macaca, Pan*). Tool use by capuchins on Jicarón shares key features with the patterns of behaviour described in long-tailed macaque populations in Thailand: stone tool use occurs solely on islands and is focused on coastal resources [104]. Another similarity to long-tailed macaques is that multiple hammering techniques are used to process particular types of prey [105]; however, the observed behavioural diversity appears to be less in these capuchins than macaques. At first glance, it appears that capuchins use stone tools more for plant materials than marine invertebrates. However, the absence of direct observation in an unhabituated population and the challenges of placing camera traps in the intertidal zone (where mounting points are rare and destruction by tides and potentially dangerous narcotics traffickers is common) currently limit our ability to fully understand the scope and frequency of tool use for marine invertebrates without direct observation.

The patterns of tool use observed in capuchins on Jicarón also share many similarities with nut-cracking behaviour in *Sapajus*. Individuals habitually re-used tools and favoured a small number of repeatedly visited tool use sites to which they transported tools and materials for processing. The accumulation of debris at these tool use sites and the comparatively dry climate on Jicarón offers potential for preservation and archaeological excavation.

Gracile capuchin tool use is also similar to chimpanzees in that capuchins transport both processing material and stone tools to particular anvil sites that are re-used over time. However, tool use also appears more sporadically in space. This population of *Cebus*, several populations of long-tailed macaques in Thailand [104,106], and some populations of *Sapajus* [107] target one of the same species—*T. catappa*. This provides a unique opportunity to directly compare tool use behaviour, quantify differences in foraging efficiency, material choice and the social behaviours surrounding tool use for an identical resource across three primate species in the wild.

**Figure 8.**
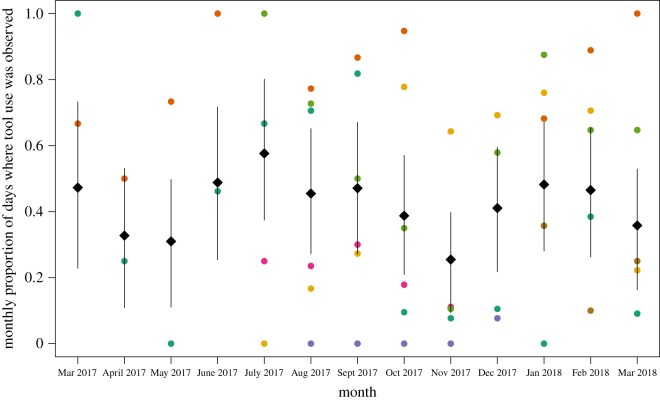
Tool use rates appear lowest in the transition months from dry to wet (April and May) and wet to dry (November) seasons. Graph shows model predictions of the probability of observing tool use per day for each month conditional on observing a capuchin at each camera site. Grey diamonds lie at posterior mean estimate while lines indicate 89% posterior credible interval. Each colour is a raw proportion per camera deployment which corresponds with a camera trap station.

Stone tool use appears to have some potential seasonality—the lowest rates of tool use occurred in the transition periods between wet and dry season (figure 8). This pattern differs from what has been observed with extractive foraging behaviours in *Cebus* in neotropical dry forests [50,72] where extractive foraging increases in the transition periods between seasons. More rigorous analysis of both behavioural and ecological data in a comparable manner is needed to verify this observation.

Similar to robust capuchins [17,28,108,109], but unlike in chimpanzees [110,111], we observe a male sex-bias in tool use (in macaques the bias differs depending on the type of tool use and food processed [112]). Despite this species having the largest degree of sexual dimorphism of any robust or gracile capuchin species and being the smallest observed wild primate species to habitually use stone tools (adult males weigh 3–3.8 kg and adult females weigh 2–3 kg [33]), we do not think this sex difference is due to differences in body size. Juveniles much smaller than adult females habitually use tools. Even if future observations do show adult female capuchins using tools, this difference is startling. Potential differences for this behaviour might be differing foraging behaviours among male and females—perhaps females are allowed access to better quality resources in fruiting trees and do not need to use stone tools to acquire adequate nutrition. Another possibility is that sex differences in the visual systems make it easier for dichromatic males to detect camouflaged almendra fruits on the forest floor compared to females who are better at detecting contrasting colours [113]. There also may be sex differences in proclivity for combinatorial actions as has been observed in *S. apella* [114]. Perhaps males are simply more likely to spend time on the ground than females as in other species of New World monkeys [115], increasing the odds that they will use tools. High population densities and resource limitation on this island might favour a reversal in philopatry from female-biased to male-biased; on a resource-limited island with high population densities, we might predict females to be the dispersing sex [116]. Thus, adult females in this group who did use tools may have emigrated to another area where we did not observe or detect them using tools and adult females we observe in this group are natal to non-tool-using groups. The presence of a localized tradition solely in the (typically) non-philopatric sex is puzzling, and something we are excited to address in the future.

### 5.1. Ecology of stone tool use

Capuchins in Coiba National Park spend an unusually high percentage of their time travelling and foraging on the ground [84], which may be due to the absence of terrestrial predators on these islands (Monteza-Moreno *et al*. [80]). By providing opportunities for capuchins to interact with and manipulate stones, this expansion into the terrestrial niche may have facilitated the emergence of this plausible cultural tradition. Additionally, the islands of Coiba National Park are particularly prone to climatic variation from El Niño–Southern Oscillation (ENSO) cycles [117], edge effects and climate change. These factors, combined with a decrease in dietary richness, might drive foraging innovation in times of resource scarcity.

It is important to note that stone tool uses on Jicarón appears to be limited to particular sections of the island. Based on our current observations, it is probably limited to a single group along the coast, despite the availability of the necessary resources in other coastal areas. We have yet to find any evidence of stone tool use on the coasts of Coiba or Ranchería (figure 1), but surveys are still underway. Further, many of the resources which capuchins on Jicarón process using stone tools are available to and eaten by other white-faced capuchin populations including hermit crabs [118], halloween crabs (B. Barrett 2017, personal observation), marine snails and coconuts [37]. Fleshy exocarps of *T. catappa* are consumed by other capuchin groups across Coiba National Park and in Costa Rica [119]—however, capuchins are unable to access the nutritious endocarps without tools. This begs the question: why does this variation exist? How does between-group and between-island dispersal structure cultural and genetic variation?

It is possible that there may exist undetected tool-using populations in other areas of Coiba National Park. As previously noted, site preservation might have an impact on the visibility of tool use but it is unlikely to obliterate all signs of such behaviour. Additionally, the rugged topography and size of the islands, limited accessibility of many locations by boat or foot and prevalence of cliffs and mangrove swamps on long stretches of coast prevent us from safely conducting randomized surveys for tool use—particularly on Coiba. Another limitation in identifying tool use on other islands is that our camera trapping effort was not proportional to island area or habitat type, as our primary aim in this initial round of research was to focus on describing a behaviour in areas and islands where we knew tool use was likely to have occurred given logistical and financial constraints. For these reasons, we have used targeted sampling and focused on looking for evidence of tool use in areas where we would predict it to exist: rocky intertidal zones with access to forest interior, areas where *T. catappa* is common, underneath trees with hard, structurally protected fruits in the forest interior and along rocky, seasonally dry stream beds. Further surveys of the forest interiors of Jicarón and Coiba and along the coast of Coiba Island are planned for future expeditions and our uncertainty of absence on coastal areas will be quantified relative to sampling effort. We hope to further increase camera trapping effort on Coiba and Jicarón in the future. We can also not rule out that ecological differences between Jicarón and populations on other islands and the mainland cannot explain the patterns of tool use we observe. Addressing this is a promising future direction, and to achieve this systematic, longitudinal data on resource availability and abundance at each site would be needed.

### 5.2. Conservation implications

How isolated populations fare is a major conservation concern, and island systems provide an opportunity to explore the impacts of long-term isolation [83] and small population size while providing insight into how rapid anthropogenic change may affect threatened mammal populations due to species invasions [120] and climate change [121,122]. The capuchins of Coiba National Park are an excellent study system to understand how geographical isolation affects both genetic and cultural diversity, and the ability of these two interacting inheritance systems to facilitate species’ responses to environmental change. Evidence of amelanism for capuchins on the island of Coiba [89] is consistent with increased homozygosity due to founder effects or inbreeding. Yet the observed behavioural flexibility of capuchins on Jicarón displayed in this paper and of capuchins on Coiba [37] suggest that they are behaviourally adapting to island life—potentially through social learning. Continued investigations into the behavioural flexibility of capuchins living in the Coiban archipelago through foraging innovation or behavioural and social flexibility will provide insight into the contributions of genetic and cultural variability in responding to ecological stress in a primate population.

One noticeable observation, is that some coastal populations of capuchins in Coiba National Park, including the one at the tool use sites, can be exposed to tremendous amounts of anthropogenic plastic waste which washes ashore from the Pacific ocean (electronic supplementary material, video S1). While primates occasionally appear to search for embedded foods in this debris, as they would browse for embedded arthropods in the leaf litter, how anthropogenic marine plastic that washes ashore impacts mammalian health and behaviour is a potentially important avenue of inquiry to examine in Coiba National Park.

### 5.3. Taxonomic status

Lastly, evaluating the taxonomic status of capuchin monkeys living in Coiba National Park is essential. Knowing whether these capuchins are an endemic species or subspecies will be important in affecting conservation decisions in an area that is threatened by development and climate change [90]. Coiba is home to an endemic subspecies of howler monkey, *A. palliata coibensis* [123,124], which some consider to be its own species, *A. coibensis* [92,125]. However, no similar genetic or morphological comparisons of Coiban archipelago versus mainland capuchin populations have been undertaken to determine if the Coiban capuchins are taxonomically unique. Capuchin samples collected in Coiba National Park in the early twentieth century were smaller than mainland samples ([126]; pp. 232–233), an observation consistent with the ‘island rule’—that larger mammals tend to become smaller on islands to cope with resource limitation [127,128]. Analysis of the genetic diversity of capuchins living across the archipelago and those of the nearby Panamanian mainland will help address taxonomic status and permit us to understand if social systems flexibly respond to the ecological pressures associated with island living. Conserving behavioural variation of cultural traditions in capuchins is also important to inform conservation decisions in Coiba National Park, as culture is an important means by which organisms adapt to environmental change [129–131]. Panama is also an important biogeographic corridor in the evolution of *Cebus*, yet there is a dearth of *Cebus* genetic samples from areas outside of the canal zone. Comparing genetic samples from Coiba National Park to more samples across the Panamanian mainland will help clarify whether Pacific capuchins should be considered a separate species (*C. imitator*) from their Atlantic congeners (*C. capucinus*) [16,45,132].

## Supplementary Material

Supplemental Material

## Supplementary Material

stone-tool-gracile supp 2

## Supplementary Material

Supplemental Video

## Data accessibility

All corresponding data and code for graphing and analysis can be found here: https://github.com/bjbarrett/CoibaCebusTool1.
