# Supplementary material for: Habitual stone-tool-aided extractive foraging in white-faced capuchins, Cebus capucinus
Source: R Soc Open Sci. 2018 Aug 22;5(8):181002. doi: 10.1098/rsos.181002 (PMC6124021; doi:10.1098/rsos.181002)
Supplement: Supplemental Material [file rsos181002supp1.pdf]

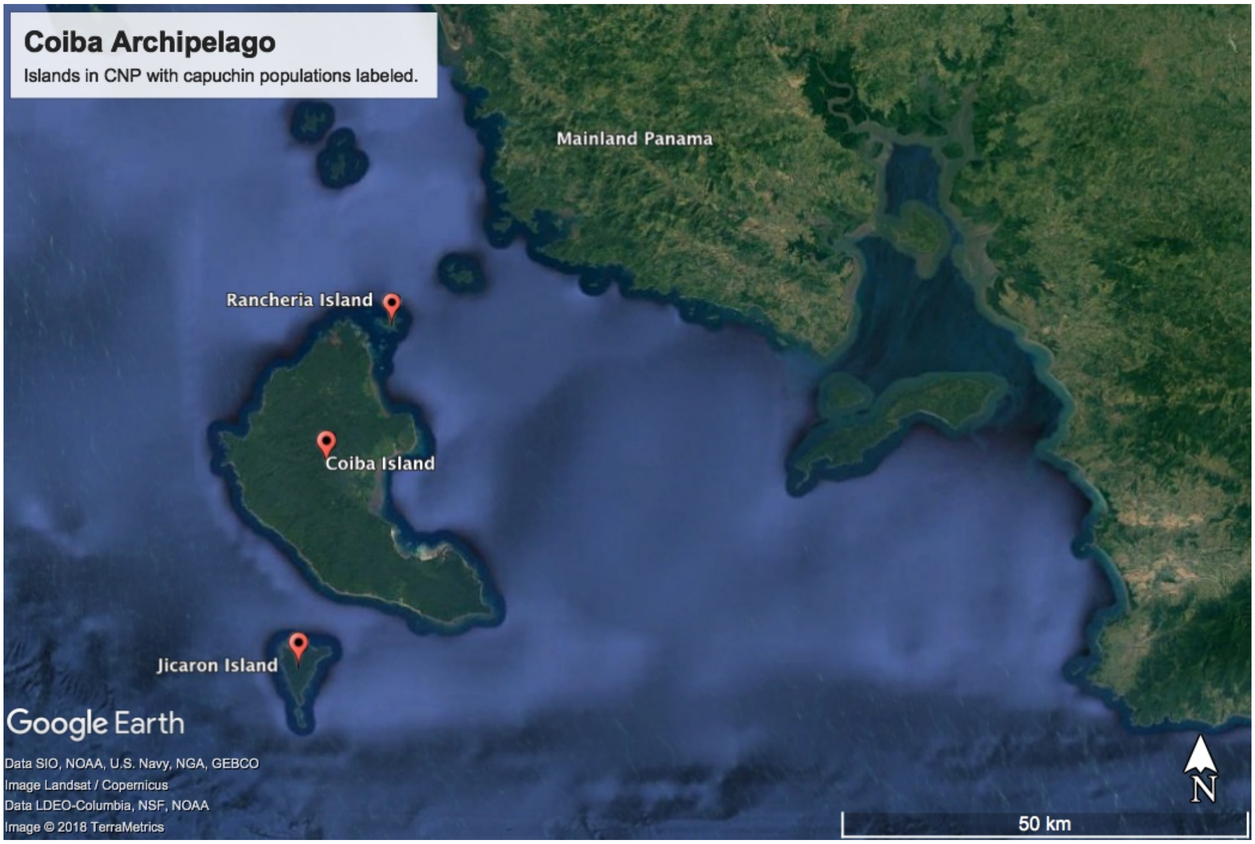

Figure S1. Map of the Coiba Archipelago off the Pacific coast of Panama. The three islands hosting populations of capuchin monkeys in Coiba National Park are labeled.

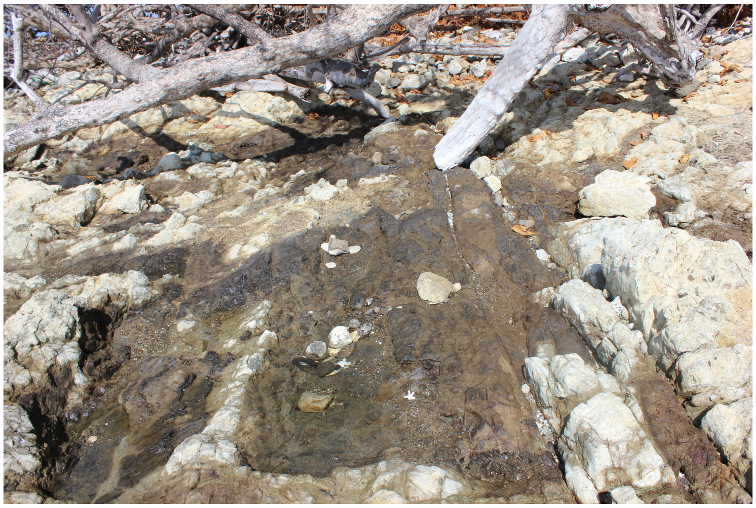

Figure S2. Recently broken clamshells at edge of intertidal zone with stones piled on them. Stone and clam accumulations spaced at distances consistent with between individual capuchin foraging proximities. We are unsure if capuchins are responsible for this or if this is a natural accumulation from tidal processes.

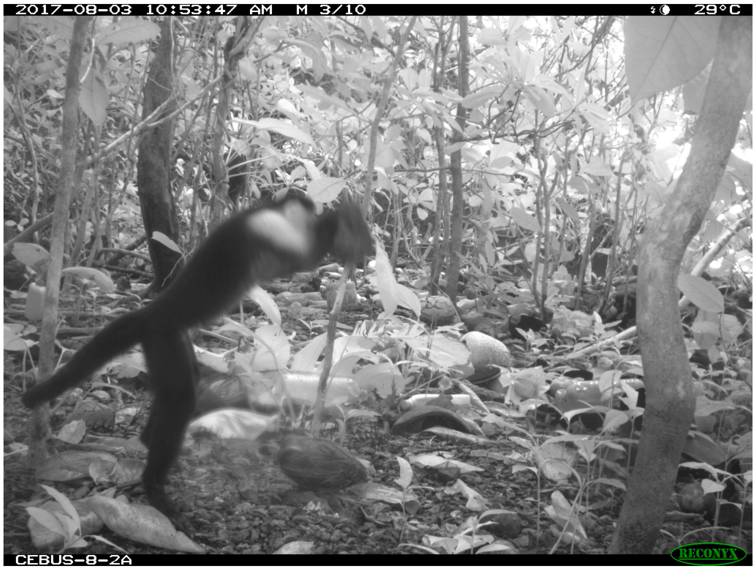

Figure S3. A juvenile male capuchin monkey breaking open a coconut husk with a hammerstone on a stone anvil.

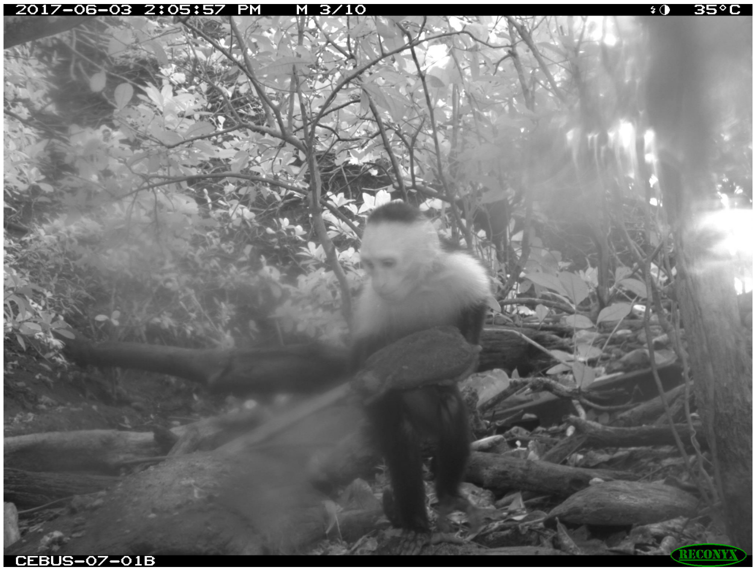

Figure S4. A juvenile male capuchin monkey transporting a stream cobble to a wooden anvil on the forest edge.

Table S1. Model parameter predictions for monthly tool use rates. Varying effects are offset form overall intercept, a.

**Parameter**	**Posterior Mean**	**Posterior SD**	**89% PCI**	
$a_{Mar2017}$	0.05	0.59	-0.86	0.97
$a_{April2017}$	-0.64	0.56	-1.51	0.23
$a_{May2017}$	-0.72	0.51	-1.50	0.11
$a_{Jun2017}$	0.13	0.50	-0.64	0.94
$a_{Jul2017}$	0.51	0.45	-0.18	1.25
$a_{Aug2017}$	-0.03	0.37	-0.59	0.57
$a_{Sep2017}$	0.04	0.38	-0.51	0.69
$a_{Oct2017}$	-0.32	0.36	-0.83	0.29
$a_{Nov2017}$	-0.98	0.41	-1.61	-0.32
$a_{Dec2017}$	-0.23	0.39	-0.86	0.35
$a_{Jan2018}$	0.09	0.39	-0.50	0.69
$a_{Feb2018}$	0.02	0.40	-0.58	0.65
$a_{Mar2018}$	-0.45	0.39	-1.07	0.19
$a_{Cebus-07-01}$	-0.45	0.60	-1.38	0.46
$a_{Cebus-07-02}$	1.79	0.68	0.76	2.86
$a_{Cebus-15-01}$	-2.12	0.96	-3.65	-0.58
$a_{Cebus-16-01}$	-0.82	0.73	-2.00	0.28
$a_{Cebus-17-01}$	0.69	0.64	-0.42	1.61
$a_{Cebus-17-02}$	0.52	0.64	-0.48	1.46
$a_{Cebus-16-02}$	-0.70	0.76	-1.89	0.46
$a_{Cebus-07-01A}$	0.05	0.50	-0.72	0.85
$a_{Cebus-07-01B}$	-0.10	0.47	-0.90	0.59
$a_{Cebus-07-01C}$	-0.63	0.53	-1.43	0.20
$a_{Cebus-07-02A}$	-0.22	0.54	-1.09	0.58
$a_{Cebus-07-02B}$	0.07	0.56	-0.76	0.99
$a_{Cebus-07-02C}$	0.00	0.55	-0.83	0.91
$a_{Cebus-15-01B}$	-0.57	0.70	-1.63	0.48
$a_{Cebus-16-01B}$	-0.34	0.58	-1.26	0.54
$a_{Cebus-16-02C}$	-0.31	0.57	-1.22	0.58
$a_{Cebus-17-01B}$	-0.53	0.51	-1.32	0.27
$a_{Cebus-17-01C}$	0.29	0.56	-0.58	1.16
$a_{Cebus-17-02B}$	-0.15	0.51	-0.90	0.67
$a_{Cebus-17-02C}$	-0.10	0.51	-0.93	0.70
a	-0.19	0.25	-0.56	0.19
σ_{month}	0.59	0.22	0.24	0.89
$\sigma_{camstat}$	1.60	0.66	0.67	2.53
$\sigma_{camstatdeploy}$	0.56	0.31	0.07	0.93

Table S2. Model parameter predictions of Gamma GLM with a log-link.

**Parameter**	**Posterior Mean**	**Posterior SD**	**89% PCI**	
a	6.51	0.05	6.44	6.58
$scale$	88.29	8.36	74.43	100.69
