# Supplementary figures and images for: Habitual stone-tool-aided extractive foraging in white-faced capuchins, Cebus capucinus
Source: R Soc Open Sci. 2018 Aug 22;5(8):181002. doi: 10.1098/rsos.181002 (PMC6124021; doi:10.1098/rsos.181002)

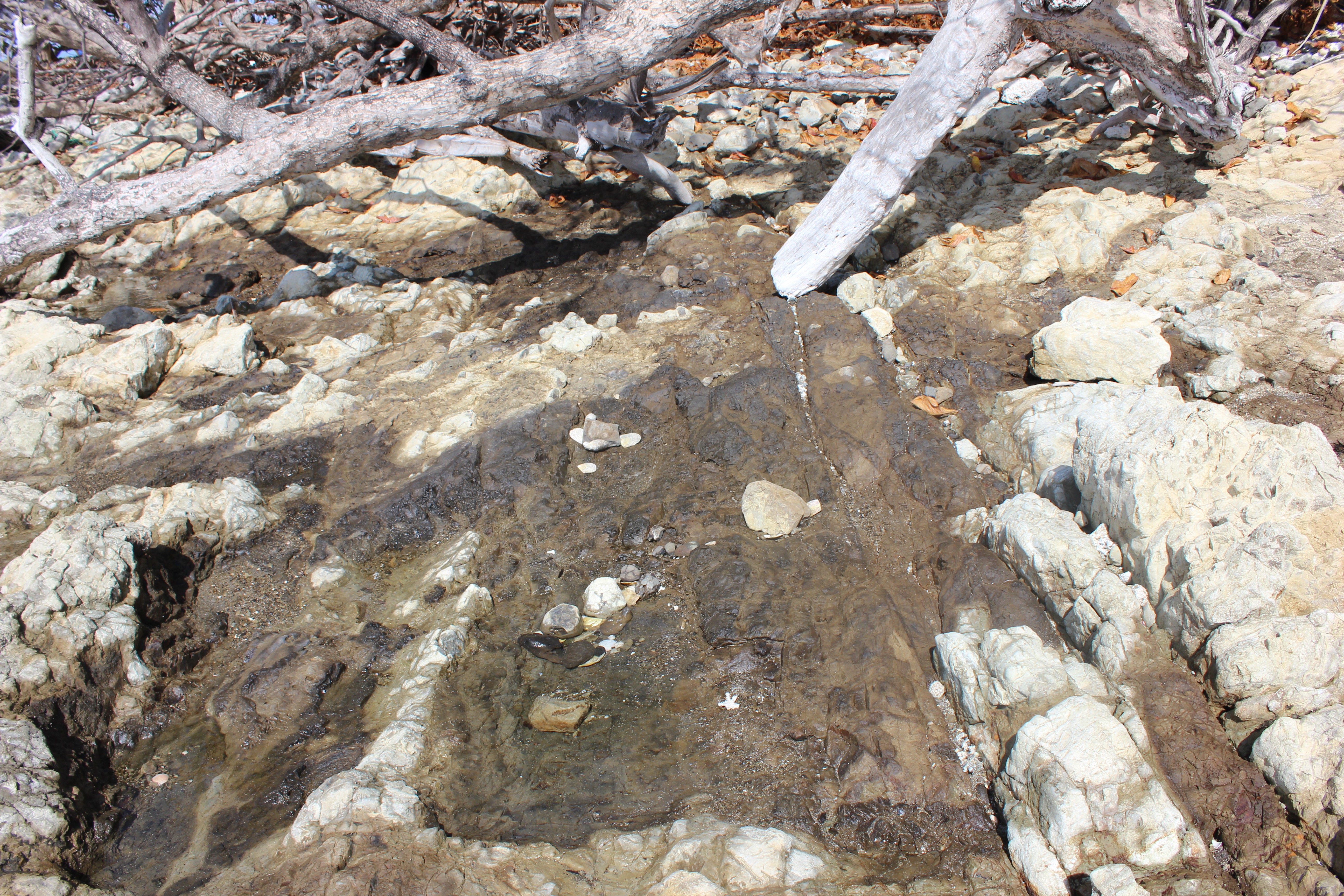

Supplement: stone-tool-gracile supp 2 [file rsos181002supp2.zip › stone-tool-gracile supp/20170327_074639_Canon_EOS_REBEL_T3i.jpg]

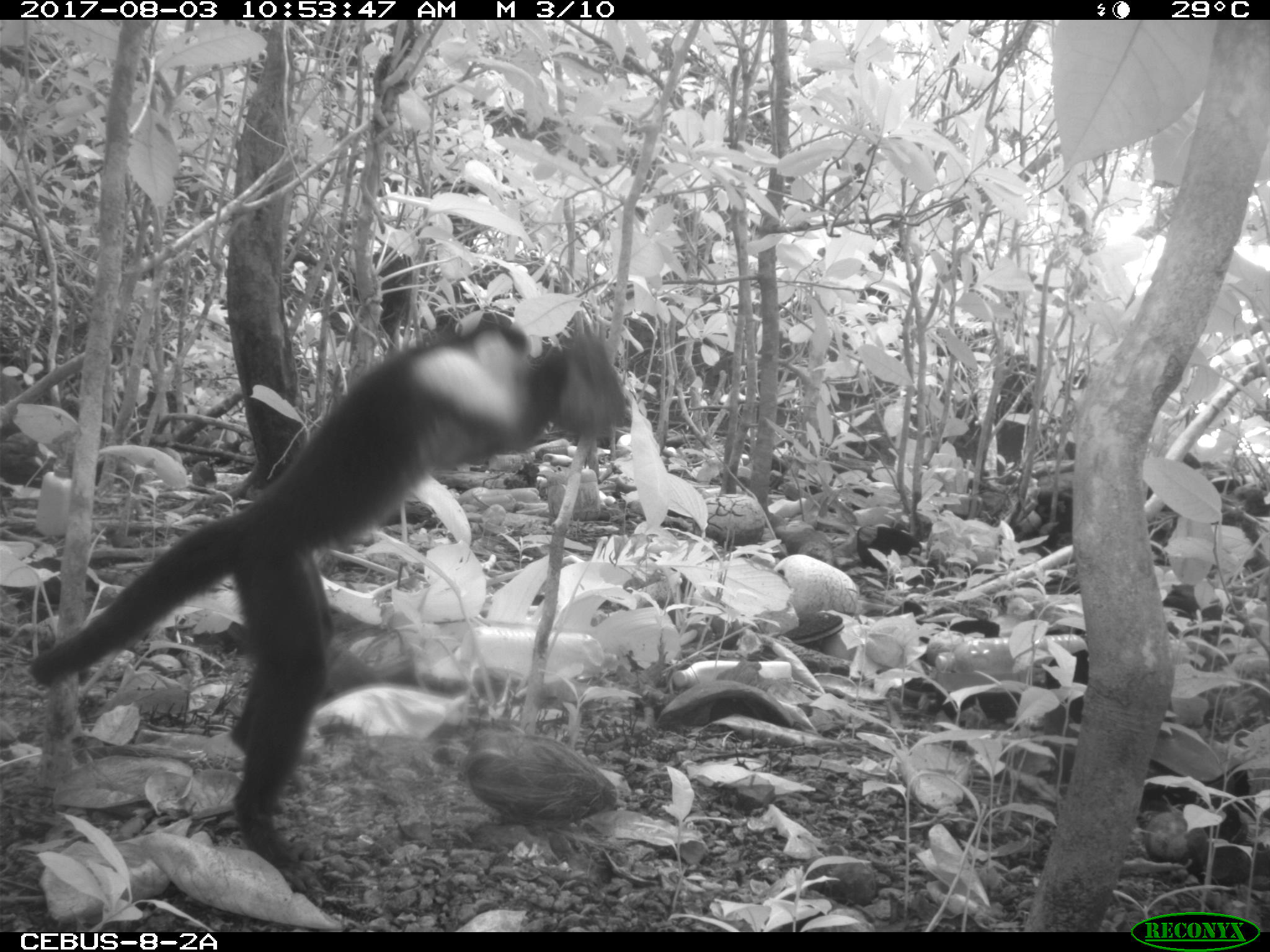

Supplement: stone-tool-gracile supp 2 [file rsos181002supp2.zip › stone-tool-gracile supp/IMG_0652.JPG]

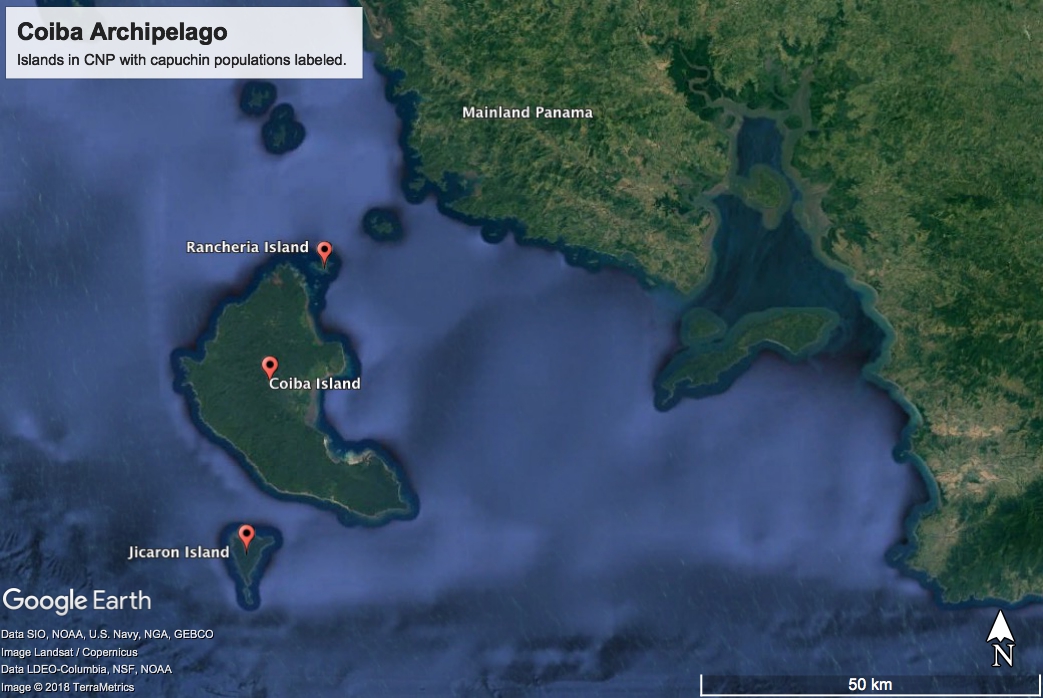

Supplement: stone-tool-gracile supp 2 [file rsos181002supp2.zip › stone-tool-gracile supp/map_of_CNP.jpg]

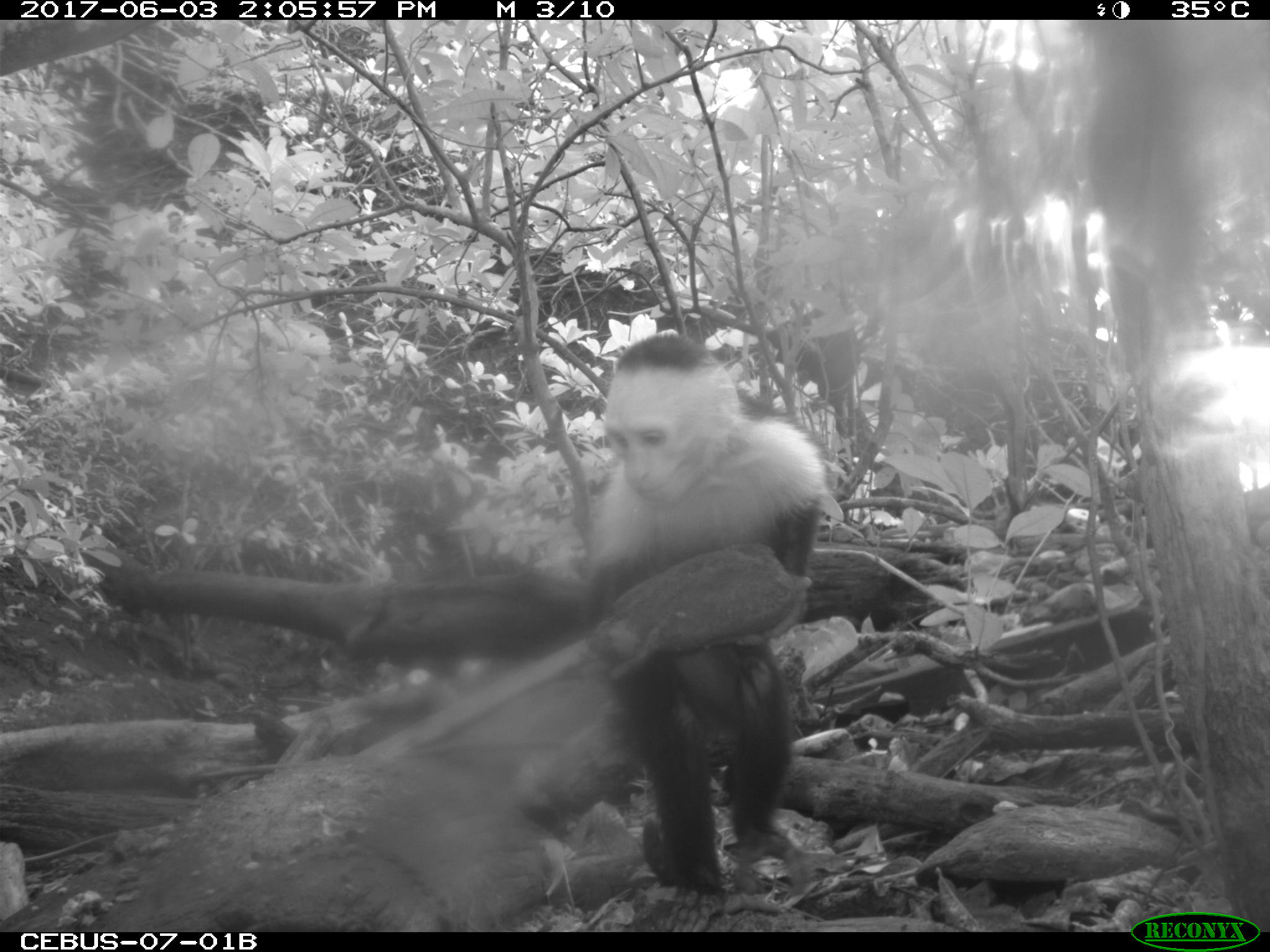

Supplement: stone-tool-gracile supp 2 [file rsos181002supp2.zip › stone-tool-gracile supp/tooltransport.JPG]
